# Annual Commencement of the Dental Department of the University of Maryland

**Published:** 1884-03

**Authors:** 


### EDITORIAL, ETC.

ANNUAL COMMENCEMENT OF THE DENTAL DEPARTMENT OF THE UNIVERSITY OF MARYLAND.—The Second Annual Commencement of this Dental Department in connection with the Seventy-Seventh Annual Session Commencement of the Medical Department of the University, was held March 14th, 1884, at the Academy of Music. The exercises were opened with prayer by the Rev. Robert H. Paine, of Mt. Calvary Church. After the reading of the mandamus, the names of the dental graduates, thirty-six in number, were announced by Prof. F. J. S. Gorgas, Dean of the Dental Department. The degree of "Doctor of Dental Surgery" was then conferred upon the following gentlemen by Hon. S. Teackle Wallis, L. L. D., Provost of the University.

| | |
|---|---|
| Theodore W. Albright, | *New York.* |
| James B. Bigham. | *South Carolina.* |
| Charles B. Blubaugh, M. D. | *West Virginia.* |
| Wilbur C. Bressler, | *Pennsylvania.* |
| Henry H. Boswell, | *Maryland* |
| John H. Brown, | *Ohio.* |
| George Buttler, Jr. | *New Jersey.* |
| John M. Comegys, | *Tennessee.* |
| George V. Copp, | *Virginia.* |
| Almond J. Cutting, | *Massachusetts.* |
| Isaac H. Davis, | *Maryland* |
| Watson E. Dorchester, | *New York.* |
| Richard D. Evans, | *S. Wales, G. B.* |
| Claus Henry Filter, | *Germany.* |
| Nathan E. Foote, | *New York.* |
| Frank C. Gallup, | *Connecticut.* |
| James Edwin Harris, | *Maryland* |
| S. Dwight Hodge, | *Vermont.* |
| R. Dallas Kibler, | *Virginia.* |
| Wiley S. Killingsworth, | *South Carolina* |
| Charles J. Ladson, | *Dist. of Columbia* |

| | |
|---|---|
| Clareuce E. Lemley, | *Virginia.* |
| William Edward Lewis, | *Florida.* |
| Job B. Mallott, | *Pennsylvnaia.* |
| Charles H. McDowell | *North Carolina.* |
| Semoney J. Minghini, | *West Virginia.* |
| S. Latimer Phillips, | *Virginia.* |
| George Edward Purnell, | *Maryland.* |
| Nelson T. Shields, | *Texas.* |
| A. La Fayette Stratford | *North Carolina.* |
| Reading B. Swindell, | *North Carolina* |
| John E. Taggart, | *Vermont.* |
| Robert R. Vaughan, | *Missouri.* |
| Richard Van der Hoppe, | *Austria.* |
| Joseph T. Wayman, | *Virginia.* |
| Elmer J. Wisherd. | *Maryland.* |

The "University pledge" relating to empiricism, was assented to by every graduate.

The following prizes were awarded by the Provost of the University:

The large and elegant "University Gold Medal,'" to Nelson T. Shields, of Texas, for the highest number of votes at the final examinations.

*Honorable Mention:* Almond J. Cutting, of Massachusetts.

*The S. S. White Prize*—"Set of Varney Plugger's," to S. Dwight Hodge, of Vermont, for the best two fillings, one of cohesive and one of non-cohesive gold.

*The S. S. White Prize*—"Dental Engine," to George E. Purnell, of Maryland for the best artificial denture on metal.

*Honorable Mention:* Nelson T. Shields, of Texas.

*The Snowden & Cowman Prize*—"Set of Harris' Forceps," to John E. Taggart, of Vermont, for the second best artificial denture on metal.

*Honorable Mention:* Wiley S. Killingsworth, of South Carolina.

*The F. L. Harris Prize.*—"Gold Medal," to A. La F. Stratford, of North Carolina, for best single non-cohesive gold filling.

*Honorable Mention:* First, Charles H. McDowell, of North Carolina, second, James B. Bigham, of South Carolina.

*The Gorgas Prize*—"Gold Medal," to James Edwin Harris, of Maryland, for best single cohesive gold filling.

*Honorable Mention:* Robert R. Vaughan, of Missouri.

*The B. H. Catching Prize.*—"*Southern Dental Journal* for one year," to Charles J. Ladson, of District of Columbia.

*The D. Genese Prize.*—"Dental Lathe, etc," to Theodore W. Albright, of New York, for best "continuous gum" artificial denture.

*The B. M. Wilkerson Prize.*—"Taft's Operative Dentistry," to Watson E. Dorchester, of New York, for best specimen tooth Filling.

*The D. Genese Prize.*—"Instrument Stand, etc," to Charles J. Ladson, of District of Columbia, for best celluloid artificial dentures.

The Valedictory Address was delivered by Hon. J. Randolph Tucker of Congress.

It was remarkably strong, and contained much solid and good advice to the graduates. He spoke for nearly one hour, and held the attention of his audience the entire time. During his remarks he said that the ambition of the young doctor must not be to rise to the highest round of the ladder only to make money, but to wear these honors well, and be honorable in wearing them. The way to secure this was only by following the maxim, "Nothing to be won in this world except by hard work." He advised the graduates to keep themselves in a school of self-training, and to train themselves in the great mysteries of their profession. Do not go out of your profession, and whatever you do, eschew politics. There are now too many political doctors. Another point is the concentration of effort and study on your one profession. This does not lead you to shun all other pleasures, but everything else must be auxiliary to the one great object most admissible. The exercises closed with the benediction.

The number of matriculates for the session was eighty-six, all of whom were purely dental students, and whose names and addresses will appear in the annual catalogue.

The University Alumni Banquet took place the evening of commencement day, at the Eutaw House, at which there was a large attendance including both the medical and dental graduates, many of the prominent physicians and dentists of the city and Maryland, adjoining States, and also the faculties of the Medical, Law and Dental Departments of the University.

The following gentlemen acted as committees in awarding the prizes (except the University Prize) at the "Prize Contest" of the graduating class of 1884, which took place in the Dental Infirmary, March 11th, 1884, and occupied the entire day. Drs. D. McFarlan, W. W. Evans, W. S. Harban and E. B. Bliss, of Washington D. C., G. H. Chewning, R. S. Switzer, and E. T. Wayman, of Virginia, B. M. Wilkerson, W. A. Mills. and A. C. McCurdy of Maryland, who expressed their admiration in the highest terms concerning the proficiency displayed by all, without a single exception of the competing students, and congratulated the Faculty of the Dental Department upon the skill and ability of the graduating class of 1884.

